# Severe Acute Respiratory Syndrome Coronavirus-2 seroprevalence in South-Central Uganda, during 2019–2021

**DOI:** 10.1186/s12879-022-07161-4

**Published:** 2022-02-21

**Authors:** Charles Ssuuna, Ronald Moses Galiwango, Edward Nelson Kankaka, Joseph Kagaayi, Anthony Ndyanabo, Godfrey Kigozi, Gertrude Nakigozi, Tom Lutalo, Robert Ssekubugu, John Bosco Wasswa, Anthony Mayinja, Martina Cathy Nakibuuka, Samiri Jamiru, John Baptist Oketch, Edward Muwanga, Larry William Chang, Mary Kate Grabowski, Maria Wawer, Ronald Gray, Mark Anderson, Michael Stec, Gavin Cloherty, Oliver Laeyendecker, Steven James Reynolds, Thomas C. Quinn, David Serwadda

**Affiliations:** 1 Rakai Health Sciences Program, P.O. Box 279, Kalisizo, Uganda; 2 Makerere University School of Public Health, Kampala, Uganda; 3 Uganda Virus Research Institute, Entebbe, Uganda; 4 Kyotera District Health Office, Kyotera District Local Government, Ministry of Health, Kyotera, Uganda; 5 Department of International Health, Johns Hopkins Bloomberg School of Public Health, Baltimore, MD USA; 6 Department of Epidemiology, Johns Hopkins Bloomberg School of Public Health, Baltimore, MD USA; 7 Division of Infectious Disease, Division of Medicine, Johns Hopkins School of Medicine, Baltimore, MD USA; 8 Abbott Laboratories, Abbott Diagnostics Division, Abbott Park, IL USA; 9 Division of Intramural Research, National Institute of Allergy and Infectious Diseases, National Institutes of Health, Bethesda, MD USA

**Keywords:** SARS-CoV-2 seroprevalence, Healthcare workers, COVID-19, South-Central Uganda

## Abstract

**Background:**

Globally, key subpopulations such as healthcare workers (HCW) may have a higher risk of contracting SARS-CoV-2. In Uganda, limited access to Personal Protective Equipment and lack of clarity on the extent/pattern of community spread may exacerbate this situation. The country established infection prevention/control measures such as lockdowns and proper hand hygiene. However, due to resource limitations and fatigue, compliance is low, posing continued onward transmission risk. This study aimed to describe extent of SARS-CoV-2 seroprevalence in selected populations within the Rakai region of Uganda.

**Methods:**

From 30th November 2020 to 8th January 2021, we collected venous blood from 753 HCW at twenty-six health facilities in South-Central Uganda and from 227 population-cohort participants who reported specific COVID-19 like symptoms (fever, cough, loss of taste and appetite) in a prior phone-based survey conducted (between May and August 2020) during the first national lockdown. 636 plasma specimens collected from individuals considered high risk for SARS-CoV-2 infection, prior to the first confirmed COVID-19 case in Uganda were also retrieved. Specimens were tested for antibodies to SARS-CoV-2 using the CoronaChek™ rapid COVID-19 IgM/IgG lateral flow test assay. IgM only positive samples were confirmed using a chemiluminescent microparticle immunoassay (CMIA) (Architect AdviseDx SARS-CoV-2 IgM) which targets the spike protein. SARS-CoV-2 exposure was defined as either confirmed IgM, both IgM and IgG or sole IgG positivity. Overall seroprevalence in each participant group was estimated, adjusting for test performance.

**Results:**

The seroprevalence of antibodies to SARS-CoV-2 in HCW was 26.7% [95%CI: 23.5, 29.8] with no difference by sex, age, or cadre. We observed no association between PPE use and seropositivity among exposed healthcare workers. Of the phone-based survey participants, 15.6% [95%CI: 10.9, 20.3] had antibodies to SARS-CoV-2, with no difference by HIV status, sex, age, or occupation. Among 636 plasma specimens collected prior to the first confirmed COVID-19 case, 2.3% [95%CI: 1.2, 3.5] were reactive.

**Conclusions:**

Findings suggest high seroprevalence of antibodies to SARS-CoV-2 among HCW and substantial exposure in persons presenting with specific COVID-19 like symptoms in the general population of South-Central Uganda. Based on current limitations in serological test confirmation, it remains unclear whether seroprevalence among plasma specimens collected prior to confirmation of the first COVID-19 case implies prior SARS-CoV-2 exposure in Uganda.

## Background

It is over a year since the Severe Acute Respiratory Syndrome Coronavirus 2 (SARS-CoV-2) emerged [1] as a global pandemic and as of the 2nd of August 2021, nearly two hundred million cases were reported globally with > 4,000,000 fatalities [2]. Transmission occurs by respiratory droplets, aerosols, and via fomites and is higher in confined or congested spaces [3]. SARS-CoV-2 infection can be asymptomatic [4] with estimates ranging from 5 to 80% while symptoms are largely nonspecific and include features of flu-like illness [5]. Diagnosis of asymptomatic and mild cases may be missed due to prioritization of screening/confirmatory tests for individuals with moderate to severe symptoms. However, asymptomatic and pre-symptomatic persons can be highly contagious and contribute greatly to epidemic spread [6, 7].

As of the 3rd of August 2021, more than 94,000 cases with 2710 deaths were documented in Uganda [2]. Between October-December 2020, the second wave of community transmission was on the rise [8] dominated by the delta variant, despite earlier control measures that included a phased nationwide lockdown between March and August 2020 [9]. The SARS-CoV-2 diagnostic testing landscape in Uganda prioritizes testing for symptomatic persons. It is unknown how many infected asymptomatic persons are missed due to this symptom-based testing approach and how this impacts community transmission.

HCW in particular are at a higher risk of contracting SARS-CoV-2 [10, 11] and inadvertently transmitting it to their patients, some of whom may be immunocompromised. According to the World Health Organization (WHO), they account for 10% of the global SARS-CoV-2 burden [12]. This risk may be higher in countries like Uganda, due to shortage of Personal Protective Equipment (PPE) amidst unquantified community disease burden. Notably, several HCW in Uganda have been infected and a number have died [13].

Due to the limited testing capacity, there are likely to be many undetected community infections fueling the epidemic. It is also unknown if SARS-CoV-2 importation or exposure in Uganda might have occurred earlier than the first (official) case reported on the 21st of March 2020. We aimed at determining the prevalence of antibodies to SARS-CoV-2 among selected high-risk sub-populations in South-Central Uganda, including HCW, persons who previously reported specific Coronavirus disease 2019 (COVID-19) like symptoms (fever, cough, loss of taste and smell) in the preceding 30 days, between May and August 2020. Additionally, we aimed at exploring the possibility of prior SARS-CoV-2 importation/exposure in South-Central Uganda before confirmation of the first (official) case on the 21st of March 2020.

## Methods

### Study design and setting

This cross-sectional study was conducted at the Rakai Health Sciences Program (RHSP) with participants recruited from within and outside the Rakai Community Cohort Study (RCCS) in four districts of South-Central Uganda (Masaka, Kyotera, Rakai and Lyantonde). The RCCS is an open, population-based cohort in 40 communities in these districts with surveys conducted ~ every 18 months among ~ 23,000 adults, resident in fishing, agrarian, or peri-urban/trading community settings [14].

### Study population and sample size

#### Healthcare workers (HCW)

A total of 385 HCW would yield an estimated prevalence of ~ 50% with 5% precision and 80% power. Assuming a 60% response rate, 642 HCW would be needed and a total of 753 HCW participated. HCW were identified from twenty-six health facilities in the region between 30th of November 2020 and 8th of January 2021, prioritizing high volume facilities located near the Uganda-Tanzania border or along the Kampala-Mutukula highway serving mobile persons who may be at higher risk of SARS-CoV-2 acquisition. At the selected health facilities, all available, willing HCW were recruited into the study.

#### Cohort participants

115 participants would yield an estimated prevalence of ~ 5% in the general population at the time with at least 4% precision (a detectable difference from zero) and 80% power. Assuming a 50% response rate, a total of 230 participants was required versus the 227 who participated—we prioritized cohort participants who previously reported COVID-19 like symptoms (fever, cough, loss of taste and/or loss of smell) in the preceding 30 days during an earlier phone-based survey conducted between May and August 2020. Blood samples from these participants were collected between 30th of November 2020 and 8th of January 2021.

#### Pre-COVID-19 response samples (before first reported case in Uganda)

595 participant samples would be required to estimate a prevalence of ~ 1% with at least 0.8% precision (a detectable difference from zero) and 80% power. We retrieved 636 archived plasma specimens collected between October 2019 and March 18th, 2020 during the preceding survey round of the RCCS, before the first confirmed COVID-19 case in Uganda was reported. These samples are routinely collected and stored primarily for surveillance of HIV, as well as genetic studies of interest in future. We prioritized samples from “high-risk” participants (truck drivers, fisher folks, commercial sex work clients, traders/vendors, shopkeepers, bar owners/workers, mechanics, and motorcycle riders)—particularly individuals living close to the Tanzanian border and along the Kampala-Mutukula highway due to their high mobility and interaction with cross-border populations.

### Sample/data collection

#### Healthcare workers

After consenting and before collection of 4 ml of venous blood, a questionnaire was administered capturing data on demographics, occupation, previous known exposure to a COVID-19 patient, self-reported prior COVID-19 PCR testing results if any and use of Personal Protective Equipment among those with prior contact with a confirmed COVID-19 case. We did not collect data on specific testing dates for those that reported prior PCR tests. 

#### Cohort participants

Following the first lockdown in Uganda on March 18th, 2020, the routine in-person RCCS survey was temporarily modified to a phone-based survey, with added emphasis on knowledge about COVID-19, mobility, and symptoms. No blood samples were collected at the time. We re-contacted individuals, who had reported the symptoms of interest, to avail 4 ml of venous blood collected in EDTA vacutainers for use in this evaluation (for those that consented). The whole blood was processed on the same day to yield plasma stored at − 80 °C till testing. 

#### Pre-COVID-19 response samples

The routine RCCS questionnaire had been administered and routine blood samples collected, which provided plasma specimens and metadata for the retrospective assessment. Plasma was frozen (− 80 °C) until laboratory analysis.

### Laboratory analysis

Frozen plasma was thawed and tested for antibodies to SARS-CoV-2 using the CoronaChek™ rapid COVID-19 IgM/IgG lateral flow test assay that uses the spike receptor binding domain (RBD) as the target antigen, following manufacturer’s instructions. We did not encounter any invalid results that would require repeat testing (as indicated by the test kit manufacturer). This assay was previously validated with Ugandan samples, including 1077 pre-pandemic samples from the RCCS [15] with sensitivity at 81.1% (CI: 64.4–91.0) for IgM, 60.5% (CI: 44.7–74.3) for IgG and 83.0 (CI: 68.9–91.5) for IgM/IgG combined while specificity was 100% (CI: 96.2–100.0) for IgM, IgG and IgM/IgG combined. Nevertheless, since low specificities of SARS-CoV-2 antibody assays have been reported, particularly from other malaria endemic regions [16, 17], any sample that was solely IgM positive by CoronaChek™ was retested by the Abbott ARCHITECT AdviseDx SARS-CoV-2 IgM chemiluminescent microparticle immunoassay (CMIA) (Abbott, Chicago, IL) to minimize false positivity. The reported positive predictive value of the AdviseDx SARS-CoV-2 IgM assay is 95% between 15 and 30 days post infection in confirmed cases, specificity is 99.56% among pre-COVID-19 samples, and within-lab precision of the assay is < 3% Coefficient of Variation.

### Data analysis

SARS-CoV-2 exposure was defined as either IgM confirmed by the ARCHITECT CMIA assay, both IgM and IgG, or IgG sole positivity. Point prevalence and 95% confidence intervals were determined for each sub-group using the exact Clopper-Pearson method of calculating confidence intervals for binomial proportions. Overall seroprevalence in each participant group was adjusted for test performance [18]. Association with seroprevalence was evaluated using chi-square tests or fisher-exact tests with a 5% level of significance. Analyses were performed in R version 4.1.2.

## Results

### Healthcare workers’ SARS-CoV-2 antibody test results

Most of the participants were female (64.54%), median age was 32 years (IQR 18–71 years), and over one-third (38.1%) were nurses. In the initial screening using the CoronaChek™, 30.8% (232/753) of HCW had detectable SARS-CoV-2 antibodies irrespective of isotype class. Of these, 119 tested positive for IgM only, 102 for both IgM and IgG, and 11 for IgG only. Of the initially 119 IgM only reactive samples, 46 were confirmed positive when re-tested using the ARCHITECT assay. After accounting for test performance, the overall SARS-CoV-2 antibody seroprevalence among HCW was 26.7% (~ 201/753, 95%CI: 23.5–29.8; Table 1). Majority (24/26) of the sampled health facilities had at least one healthcare worker who had antibodies to SARS-CoV-2. Seroprevalence at facilities ranged from 4.2 to 57.0% with a median of 20.0%. Seroprevalence did not differ by HCW cadre, age, or sex (Table 2).

**Table 1:** SARS-CoV-2 seroprevalence estimates, adjusting for test performance

Category	IgM_only (CoronaCheck, then Architect)	IgM_and_IgG (CoronaCheck)	IgG_only (CoronaCheck)	Overall adjusted prevalence (95% CI)^e^
HCW, (N = 753)				
Crude number + ve^a^	46	102	11	200.8/753 (26.7%, CI = 23.5–29.8)
Crude prevalence^b^	6.1%	13.5%	1.5%	
Adjusted prevalence^c^	7.9%	16.3%	2.4%	
Adjusted number + ve^d^	59.7	122.9	18.2	
Cohort participants, (N = 227)				
Crude number + ve	7	15	5	35.4/227 (15.6%, CI = 10.9, 20.3)
Crude prevalence	3.1%	6.6%	2.2%	
Adjusted prevalence	4.0%	8.0%	3.6%	
Adjusted number + ve	9.1	18.1	8.3	
Early samples, (N = 636)				
Crude number + ve	8	1	2	14.9/636 (2.3%, CI = 1.2, 3.5)
Crude prevalence	1.3%	0.2%	0.3%	
Adjusted prevalence	1.6%	0.2%	0.5%	
Adjusted number + ve	10.4	1.2	3.3	

^a^Crude number + ve are the number of participants who tested positive on that test

^b^Crude prevalence = crude number + ve/number of participants tested

^c^Adjusted prevalence = (crude prevalence + specificity − 1)/(sensitivity + specificity − 1). The sensitivity and specificity of CoronaCheck is 81.1% and 100% respectively for IgM; 60.5% and 100% respectively for IgG; and 83.0% and 100% for IgM and IgG combined. Sensitivity and specificity of the Architect assay is 95.0% and 99.56% respectively for IgM. Combined sensitivity of CoronaCheck and the Architect assay when used serially for IgM = Se1 × Se2 = 81.1% × 95.0% = 77.0%; while the combined specificity of the two assays used serially = 1 − (1 − Sp1)*(1 − Sp2) = 1 − (1 − 100%) × (1–99.56%) = 100%

^d^Adjusted number + ve = adjusted prevalence × number of participants tested

^e^Overall adjusted prevalence = (Sum of adjusted numbers + ve on IgM only, IgM and IgG, and IgG only)/number of participants

**Table 2:** Factors associated with SARS-CoV-2 seroprevalence among Healthcare workers

Sociodemographic characteristics	n (row %) seropositive Crude N = 159	n (row %) seronegative Crude N = 594	p-value
Sex			
Male	61 (22.8)	206 (77.2)	0.442
Female	98 (20.2)	388 (79.8)	
Age category			
18–24	33 (17.6)	155 (82.4)	0.444
25–34	57 (23.9)	181 (76.1)	
35–44	39 (20.2)	154 (79.8)	
45–54	21 (25.0)	63 (75.0)	
55 +	9 (18.0)	41 (82.0)	
Cadre			
Medical Officer	3 (25.0)	9 (75.0)	0.523
Clinical Officer	8 (30.8)	18 (69.2)	
Nurse (all levels)	57 (19.9)	230 (80.1)	
Lab tech (all levels)	16 (26.2)	45 (73.8)	
Other staff*	75 (20.5)	290 (79.5)	

*Cleaners, counselors, data clerks, drivers, cooks, security officers, and student nurses

A total of 128 HCW reported having undergone prior SARS-CoV-2 RT-PCR testing with 16 reporting a prior positive result. Of the 16 individuals, 8 still had detectable antibodies to SARS-CoV-2 (1 with IgM only, 1 with IgG only, 6 with both IgM and IgG). Out of 108 HCW who reported a previous negative RT-PCR result, 29 (27%) subsequently tested antibodies positive. Four individuals did not know their results.

### Exposure, use of Personal Protective Equipment (PPE), and seroprevalence among HCW

Twenty one percent (156/753) of the HCW surveyed reported previous contact with a confirmed COVID-19 case. All 156 reported consistently using face masks during known exposure, with a few also using other forms of PPE such as face shields, gowns, aprons, and gloves (Table 3). Despite reporting consistent use of face masks, 63/156 (40%, CI 33–49%) of the known exposed HCW had antibodies to SARS-CoV-2.

**Table 3:** Association of Personal Protective Equipment use and seroprevalence among Healthcare workers who were contacts of confirmed COVID-19 cases

	n (%) Seropositive Crude N = 45	n (%) Seronegative Crude N = 111	p-value
Face masks, n (%)			
Yes	45 (29%)	111 (71%)	
No	–	–	
Gloves, n (%)			
Yes	28 (34%)	55 (66%)	0.151
No	17 (23%)	56 (77%)	
Face shields, n (%)			
Yes	11 (29%)	27 (71%)	0.987
No	34 (29%)	84 (71%)	
Gowns, n (%)			
Yes	12 (37.5%)	20 (62.5%)	0.226
No	33 (27%)	91 (73%)	
Aprons, n (%)			
Yes	15 (33%)	31 (67%)	0.502
No	30 (27%)	80 (73%)	

### Cohort participants’ SARS-CoV-2 antibody test results

A total of 314 individuals in the prior phone-based survey had reported the symptoms of interest (cough, fever, loss of taste and loss of smell). We attempted to contact all these for participation in this current survey and recruited 227 (72%). The others were not recruited due to either refusal or failure to locate them (unreachable telephone contacts and out-migration). Of those recruited, most (69.1%) were female and median age was 39 years (IQR 20–49 years). Upon initial screening using the CoronaChek™, 16.3% of the participants (37/227) tested positive on IgM only, 2.2% (5/227) tested positive on IgG only whereas 6.6% (15/227) were positive on both IgM and IgG. Following re-testing of the initially IgM only reactive samples using the ARCHITECT assay, 7/37 were confirmed positive. After accounting for test performance, the overall SARS-CoV-2 antibody seroprevalence among symptomatic cohort participants was 15.6% (~ 35/227, 95% CI: 10.9, 20.3; Table 1). Seroprevalence did not differ by participant HIV status, sex, age, or occupation (Table 4).

**Table 4:** Factors associated with SARS-COV-2 seroprevalence among phone-based survey participants

Sociodemographic characteristics	n (row %) seropositive Crude N = 27	n (row %) seronegative Crude N = 200	p-value
HIV status			
Negative	14 (12.0)	103 (88.0)	1.000
Positive	13 (11.8)	97 (88.2)	
Sex			
Male	9 (12.9)	61 (87.1)	0.939
Female	18 (11.5)	139 (88.5)	
Age category			
20–24	0 (0.0)	10 (100)	0.633
25–34	7 (11.1)	56 (88.8)	
35–44	14 (11.9)	104 (88.1)	
45–54	6 (16.7)	30 (83.3)	
Occupation			
Agriculture for home use/barter	10 (17.9)	46 (82.1)	0.097
Agriculture for selling	1 (2.9)	34 (97.1)	
Fishing	3 (20.0)	12 (80.0)	
Shopkeeper	3 (25.0)	9 (75.0)	
Trading/vending	5 (12.2)	36 (87.8)	
Bar worker or owner	2 (33.3)	4 (66.7)	
Waitress/Waiter/restaurant owner	1 (16.7)	5 (83.3)	
Construction	1 (50.0)	1 (50.0)	
Motorcycle riders (carrying passengers)	1 (25.0)	3 (75.0)	

### SARS-CoV-2 antibody test results among plasma specimens collected prior to confirmation of the first COVID-19 case in Uganda

Upon initial screening using the CoronaChek™, 7% (47/636) of specimens had detectable antibodies to SARS-CoV-2 irrespective of isotype class. The majority (44) were positive on IgM only, 2 were positive on IgG only whereas 1 reacted for both IgM and IgG. Out of the 44 IgM sole positive samples, 8 were confirmed following re-testing using the ARCHITECT assay. After accounting for test performance, the overall SARS-CoV-2 antibody seroprevalence in these early samples was 2.3% (~ 15/636, 95%CI: 1.2, 3.5; Table 1). Of the 11 samples actually confirmed positive for IgM/IgG, 9 were collected between October and December 2019, and 2 collected between Jan-March 2020.

## Discussion

These findings suggest a relatively high SARS-CoV-2 seroprevalence among HCW at almost all the selected health facilities (24/26) in South-Central Uganda and substantial seroprevalence in persons previously reporting specific COVID-19 like symptoms within the general population. There was also potentially a spike in transmission a few weeks prior to this evaluation with predominance of IgM only antibodies in most of the participants. This is similar to findings by Nguyen et al. showing higher COVID-19 incident reports by frontline health care workers largely arising from inadequacy of PPE among other factors[19] and emphasizes need to avail adequate PPE to healthcare workers following evidence[20] that adequate PPE (and other measures) can effectively protect healthcare workers against COVID-19. HCW should also be prioritized for vaccination whenever available. Limited PPE access may enhance epidemic spread given the large numbers of clients that healthcare workers handle.

There are challenges interpreting SARS-CoV-2 rapid serology in regions with high malaria endemicity as infection with *Plasmodium* species was shown to induce cross-reactive antibodies to carbohydrate epitopes on the SARS-CoV-2 spike protein [17, 21]. It is thus unclear whether seroprevalence in plasma specimens collected prior to confirmation of the first COVID-19 case implies prior SARS-CoV-2 or other related coronavirus exposure or malaria in Uganda. One key limitation of this study is the limited (sub) populations that were evaluated which may affect generalizability at population level.

HCW are minimally protected by face masks and only a few had accesses to other PPE (face shields, gowns, aprons etc.) and this, coupled with likelihood of improper face mask use or lack of N95-level protection, could explain the positive COVD-19 antibody results observed even among participants reporting face mask use. Several undetected cases among HCW in this region is a potential driver of nosocomial spread. A moderate concordance between reported RT-PCR COVID-19 positives and antibody test outcome may reflect waning antibody levels as reported in several publications [22, 23].

## Conclusions

Findings suggest a high seroprevalence of antibodies to SARS-CoV-2 among HCW and substantial exposure in persons presenting with specific COVID-19 like symptoms in the general population of South-Central Uganda. Based on current limitations in serological test confirmation, it remains unclear whether seroprevalence in plasma specimens collected prior to confirmation of the first COVID-19 case implies prior SARS-CoV-2 exposure in Uganda.

## Data Availability

All data generated or analyzed during this study are included in this published article.

## Abbreviations

SARS-CoV-2: Severe Acute Respiratory Syndrome Coronavirus 2
HCW: Healthcare workers
COVID-19: Coronavirus disease 2019
CMIA: Chemiluminescent microparticle immunoassay
WHO: World Health Organization
PPE: Personal Protective Equipment
UNCST: Uganda National Council for Science and Technology
RCCS: Rakai Community Cohort Study
RHSP: Rakai Health Sciences Program
